# Supplementary material for: Formation of Lymphoma Hybrid Spheroids and Drug Testing in Real Time with the Use of Fluorescence Optical Tweezers
Source: Cells. 2022 Jul 5;11(13):2113. doi: 10.3390/cells11132113 (PMC9265821; doi:10.3390/cells11132113)
Supplement: Supplementary file 1 [file cells-11-02113-s001.zip › Figure S1.pdf]

Article

Supplementary Materials. Formation of Lymphoma Hybrid Spheroids and Drug Testing in Real-time with the Use of Fluorescence Optical Tweezers.

Kamila Duś-Szachniewicz , Katarzyna Gdesz-Birula , Emilia Nowosielska , Piotr Ziółkowski and Sławomir Drobczyński

Supplementary Figures

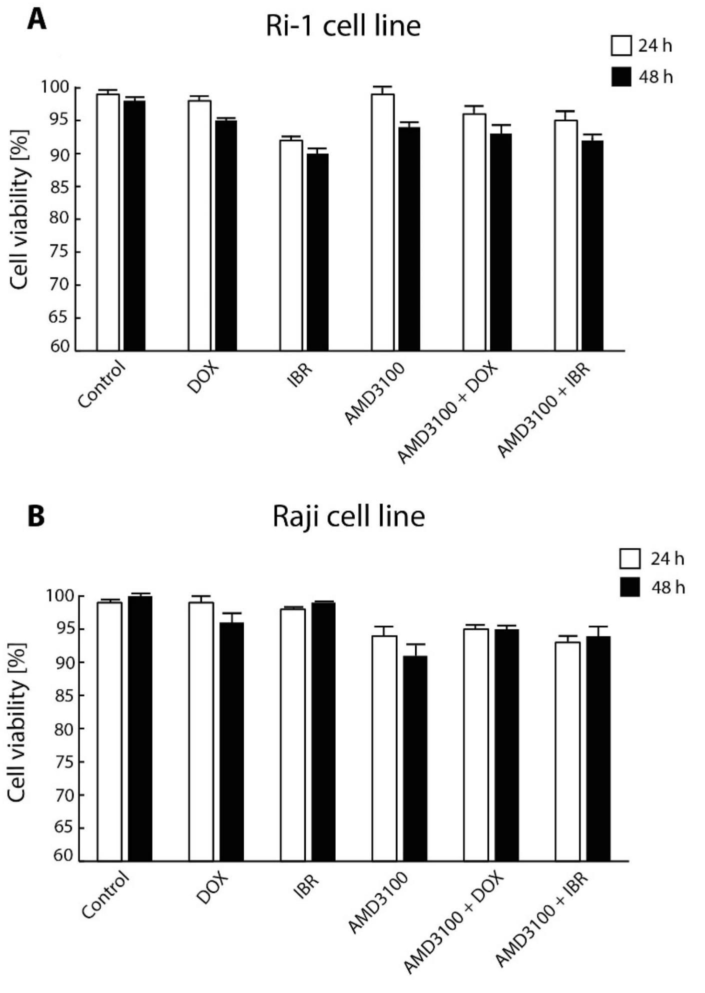

Figure S1. The effect of the anticancer treatment on the viability of Ri-1 (A) and RAJI (B) cell lines. Error bars represent the standard error of the mean calculated from three independent measurements. DOX, doxorubicin, IBR, ibrutinib.
